# Clinical and Prognostic Association of Transcription Factor SOX4 in Gastric Cancer

**DOI:** 10.1371/journal.pone.0052804

**Published:** 2012-12-20

**Authors:** Chia-Lang Fang, You-Cheng Hseu, Yi-Feng Lin, Shih-Ting Hung, Chein Tai, Yih-Huei Uen, Kai-Yuan Lin

**Affiliations:** 1 Department of Pathology, School of Medicine, College of Medicine, Taipei Medical University, Taipei, Taiwan; 2 Department of Pathology, Wan Fang Hospital, Taipei Medical University, Taipei, Taiwan; 3 Department of Cosmeceutics, China Medical University, Taichung, Taiwan; 4 Division of General Surgery, Department of Surgery, Chi Mei Hospital Chiali, Tainan, Taiwan; 5 Division of General Surgery, Department of Surgery, Chi Mei Medical Center, Tainan, Taiwan; 6 Department of Medical Research, Chi Mei Medical Center, Tainan, Taiwan; 7 Department of Biotechnology, Southern Taiwan University of Science and Technology, Tainan, Taiwan; 8 Superintendent's Office, Chi Mei Hospital Chiali, Tainan, Taiwan; 9 Institute of Biomedical Engineering, Southern Taiwan University of Science and Technology, Tainan, Taiwan; 10 Department of Biotechnology, Chia Nan University of Pharmacy and Science, Tainan, Taiwan; The Chinese University of Hong Kong, Hong Kong

## Abstract

Gastric cancer (GC) is one of the most common malignant cancers worldwide. However, little is known about the molecular process by which this disease develops and progresses. This study investigated correlations between the expression of nuclear transcription factor SOX4 and various clinicopathologic parameters as well as patients' survival. Expression levels of nuclear SOX4 were analyzed by immunohistochemistry; the data comprised gastric tissues from 168 patients with GC. Paired *t* tests were used to analyze the differences in nuclear SOX4 expression between tumor and non-tumor tissues from each patient. Two-tailed Χ^2^ tests were performed to determine whether the differences in nuclear SOX4 expression and clinicopathologic parameters were significant. Time-to-event endpoints for clinicopathologic parameters were plotted using the Kaplan-Meier method, and statistical significance was determined using univariate log-rank tests. Cox proportional hazard model was used for multivariate analysis to determine the independence of prognostic effects of nuclear SOX4 expression. Overexpression of nuclear SOX4 was significantly correlated with depth of invasion (*P*<0.0001), nodal status (*P* = 0.0055), distant metastasis (*P* = 0.0195), stage (*P* = 0.0003), and vascular invasion (*P* = 0.0383). Patients who displayed high expression levels of nuclear SOX4 achieved a significantly poorer disease-free survival rate, compared with patients with low SOX4 expression levels (*P* = 0.003). Univariate Cox regression analysis showed that overexpression of nuclear SOX4 was a clear prognostic marker for GC (*P* = 0.004). Overexpression of nuclear SOX4 can be used as a marker to predict the outcome of patients with GC.

## Introduction

The incidence of gastric cancer (GC) declined between the 1940 s and the 1980 s in the Western world, but GC remains extremely widespread throughout the world. It affects approximately one million people annually and is the second most frequent cause of cancer death [1], [2]. A wide variation in incidence is evident across the continents [1]. In Asia and parts of South America, GC is the most common epithelial malignancy and is a leading cause of cancer-related deaths. In Taiwan, government statistics issued in 2011 ranked GC as the sixth most frequently diagnosed malignant disease, resulting in more than 2000 deaths annually (http://www.doh.gov.tw/statistic/index.htm). Substantial advances have been made in surgical techniques and chemotherapy, which have improved the treatment of GC, but the cure rate for advanced cases remains low and the morbidity remains high [3], [4]. A fuller understanding of the pathogenesis and biological features of the disease is necessary to further enhance treatment methods.

Among the prognostic markers now available for GC, the most important is the American Joint Committee on Cancer (AJCC) stage determined by the depth of invasion, the involvement of the lymph nodes, and distant metastasis. However, the prognosis varies even among patients at the same stage. Thus, the search for specific biological markers to identify subgroups of patients who are likely to experience a particularly aggressive course of disease is ongoing [5]. In recent decades, several studies have suggested that genetic alterations may play a role in the development and progression of GC [6]. Studies in molecular pathology may help in understanding the disease pathogenesis and might also reveal useful prognostic molecular markers. Some suggested biological prognostic markers include overexpression of protein kinase CK2, Vav3, mesothelin, and epidermal growth factor receptor [7]–[11].

In humans, the sex-determining region Y (SRY) box family, also referred to as the SOX family, comprises 20 highly conserved transcription factors that play important roles in development. These transcription factors are defined by a conserved signature sequence in the high-mobility group (HMG) DNA-binding domain (DBD) [12], [13]. SOX4 is a 47-kDa protein that is encoded by a single exon gene, which is highly conserved in vertebrates. In mice, SOX4 is specifically expressed in the ovary, testis, mammary gland, and thymus and in mouse T and pre-B lymphocytic cell lines [14]. In addition, SOX4 is essential for the development of the heart, lymphocytes, and thymocytes, and SOX4-null mice die from cardiac defects [15]. The proliferative capacity of B-cell progenitors is severely decreased in cells from SOX4 knockout mice [16].

The clinical importance of SOX4 has gained increasing attention in recent years, with numerous reports suggesting that SOX4 may contribute to tumor progression. Three independent studies screening for important oncogenes showed that SOX4 is frequently altered through retroviral insertions [17]–[19]. The murine leukemia virus typically targeted SOX4, and stabilized the SOX4 message to produce B-cell lymphomas that displayed increased SOX4 message levels [19]. Increased SOX4 expression is associated with tumors of the bladder, prostate, and colon, and with non-small-cell lung tumors [20]–[23]. However, the role of SOX4 in such tumors is not fully understood and the reported data have shown certain contradictions. Whereas SOX4 knockdown resulted in apoptosis of ACC3 adenoid cystic carcinoma cells, SOX4 overexpression promoted cell cycle arrest and apoptosis of HCT116 colon carcinoma cells [24], [25]. The microRNA (miRNA) miR-335 inhibited metastatic cell invasion and acted, at least in part, through targeting *sox4* and its putative target *TNC*, which encodes an extracellular matrix component implicated in cell migration [26]. By contrast, the higher the level of SOX4 expression, the better the prognosis for patients with medulloblastomas and other tumor types [27]. Thus, SOX4 might exert different effects on tumor cells depending on the context and primary transformation mechanism; further studies are warranted to clarify this issue.

To date, the prognostic significance of nuclear SOX4 expression levels in human GC has not been established. This study investigated the correlations between nuclear SOX4 expression and clinicopathologic parameters, and evaluated the significance of nuclear SOX4 in predicting the prognosis for patients with GC.

## Materials and Methods

### Ethics Statement

The institutional review board at Chi Mei Medical Center approved the tissue acquisition protocol for the immunohistochemical and immunoblotting study. Written informed consent was obtained from each participant before tissue acquisition.

### Participants and Specimens

The patient cohort comprised 168 consecutive GC cases from 1997 through 2004 documenting pathologic and clinical factors and clinical outcome. All cases in this study received radical total or subtotal gastrectomy with D2 or D3 lymph node dissection. Completeness of surgical resection was achieved in all cases, and pathological examination revealed no tumor involvement of the resection margins in surgical specimens (classified as R0: no residual tumor based on R category in AJCC classification). None of our study patients had received preoperative chemotherapy and/or radiotherapy. The non-tumor portion was obtained from grossly normal gastric mucosa, separate from the tumor, in resected gastric specimen. Clinicopathologic parameters of GCs were determined according to the AJCC classification. The follow-up duration for disease-free survival was defined as the period between the operation date and the day of relapse, according to the patient's chart. For each patient, we analyzed a pair of tumor and non-tumor gastric tissues to determine the nuclear SOX4 expression.

### Immunohistochemical Analysis

Nuclear SOX4 expression was analyzed by immunohistochemistry. Paraffin-embedded tissue blocks were sectioned at 5 mm and transferred to microscope slides (Muto Pure Chemicals Co. Ltd., Tokyo, Japan). Breast tissue was used a positive control for SOX4. The negative control entailed omission of the primary antibody and incubation with phosphate buffer saline. Sections were dewaxed with xylene, followed by rehydration in graded alcohols. Deparaffinized sections were incubated in pH 6.0 citrate buffer for 40 min at 95°C on a hotplate to retrieve antigens. Further antigen blocking was performed using Dako REAL Peroxidase-Blocking Solution (Dako North America Inc., Carpinteria, CA) for 5 min. The slides were subsequently incubated with primary antibody: polyclonal anti-SOX4 (Life-Span, Victoria, Canada) for 1 hour at room temperature, at a dilution of 1∶400. Detection of the immunoreactive staining was conducted using the avidin-biotin-peroxidase complex method according to the manufacturer's instructions. A sensitive Dako REAL EnVision Detection System (Dako North America Inc., Carpinteria, CA) was used. After incubation with diaminobenzidine for 5 minutes, the sections were counterstained with hematoxylin and mounted in Dako Faramount Aqueous Mounting Medium (Dako North America Inc., Carpinteria, CA) for microscopic interpretation. As a transcription factor, only nuclear SOX4 was scored. Semiquantitative scoring of intensity (0, no staining; 1, weak staining; 2, strong staining) and fraction of positive cancer cells (0, no staining; 1, less than half; 2, more than half) was undertaken [22]. The final score was calculated for each sample by multiplying the intensity and the percentage of immunostaining: 0, no staining; 1, weak staining; 2, moderate staining; 4, strong staining. Sections with a score of 0 or 1 displayed low expression of SOX4, whereas those that scored 2 or 4 were defined as having high expression or overexpression of SOX4. Clinical data collection and immunohistochemical analysis were performed independently of each other, in an investigator-blinded study.

### RNA Extraction and cDNA Synthesis

According to the manufacturer's instructions, total RNA from 10 tumor and non-tumor pairs of gastric tissues was isolated by using an RNA extraction kit (Sigma, St. Louis, MO). RNA quality was analyzed by using Agilant 2100 Bioanalyzer. The RIN values of all 20 samples were above 7. cDNA synthesis was performed as described in our previous study [28]. Synthesized cDNA was stored at −20°C until use.

### Primers and Probes

Taqman Gene Expression Assays including primers and probes of SOX4 and β-actin, an internal control, were purchased from Applied Biosystems. The Assay numbers of SOX4 and β-actin were Hs00268388_s1, and Hs99999903_m1, respectively.

### Quantitative Real-time PCR

The expression levels of the target genes were measured using quantitative real-time PCR in the ABI Prism 7300 Sequence Detection System (Applied Biosystems) as described in our previous study [28]. Threshold cycle (*C_t_*) is the fractional cycle number at which the fluorescence generated by cleavage of the probe exceeds a fixed level above baseline. For a chosen threshold, a smaller starting copy number results in a higher *C_t_* value. The amount of SOX4 mRNA in tumor or non-tumor tissues, standardized against the amount of β-actin mRNA, was expressed as Δ*C_tumor_* or Δ*C_non-tumor_*  =  *C_t_*_(SOX4)_ – *C_t_*_(β-actin)_.

### Cell Culture

Human normal (Hs738.St/Int) and GC cell lines (AGS and NCI-N87) were obtained from the American Type Culture Collection (ATCC; Manassas, VA, USA). Cell lines were authenticated by the ATCC cell biology program, and were passaged for no longer than 6 months before new cells were brought out of the frozen state or a new cell aliquot was purchased from ATCC. Cells were cultured in DMEM (Hs738.St/Int), F-12K (AGS), or RPMI-1640 (NCI-N87) media supplemented with 10% fetal bovine serum, 100 units/mL penicillin G, 100 µg/mL streptomycin sulfate, and 250 ng/mL amphotericin B.

### Nuclear Protein Preparation

Nuclear proteins were extracted using NE-PER Nuclear Extraction Reagent (Pierce Biotechnology, Rockford, IL), according to the manufacturer's instructions. The samples were stored at −80°C until used. The protein concentration was determined using a BCA Protein Assay Kit (Pierce Biotechnology) with bovine serum albumin as a standard.

### Immunoblotting

Denatured protein samples were subjected to 12% SDS-PAGE. Proteins were transferred to nitrocellulose membranes, and blocked blots were incubated at 4°C overnight with anti-SOX4 polyclonal antibody (1∶1000 dilution). TATA binding protein was used as an internal control for equal protein loading. The cell fractionation was confirmed by β-actin to rule out the possibility of contamination. Blots were further incubated with secondary antibodies conjugated with peroxidase (Sigma, St. Louis, MO) for 1 h at room temperature. They were then incubated with SuperSignal West Femto Maximun Sensitivity Substrate (Pierce Biotechnology, Inc., Rockford, IL), and exposed to a Fuji medical x-ray film (Fuji Photo Film Co., Tokyo, Japan). Image processing was performed using Fuji Image Gauge software.

### Statistical Analysis

Paired *t* tests were used to assess the difference in nuclear SOX4 expression between tumor and non-tumor tissues for each patient. We examined several clinicopathologic parameters: age, gender, depth of invasion, nodal status, distant metastasis, stage, degree of differentiation, and vascular permeation. The correlation between nuclear SOX4 expression and each clinicopathologic parameter was examined using χ^2^ test. The time-to-event endpoints for all clinicopathologic parameters were plotted by the Kaplan-Meier method, and the degree of significance was calculated by the univariate log-rank test. Parameters that emerged as significant (*P*≤0.05) in univariate analysis were entered as variables in the multivariate Cox regression model, and hazard ratio (HR) and independence of prognostic impact could be determined in a stepwise backward fashion. All data were analyzed using SPSS software version 14 (SPSS, Chicago, IL). A *P* value of <0.05 was considered significant.

## Results

### Basic Data

This study enrolled 168 patients with GC, 104 of whom were men and 64 were women (Table 1). The patients' ages ranged from 34 to 88 years at first diagnosis (mean 64.9 years). Based on the AJCC classification, 45 patients were at stage I, 45 were at stage II, 54 were at stage III, and 24 were at stage IV. The follow-up period for all patients ranged from 0 to 136.2 months (mean 21.4 months). During follow-up, 86 patients died of GC.

**Table 1:** Demographic data and survival in different stages of GC according to the AJCC classification.

	Stage I	Stage II	Stage III	Stage IV	Total
	(n = 45)	(n = 45)	(n = 54)	(n = 24)	(n = 168)
Gender					
Male	22	32	36	14	104
Female	23	13	18	10	64
Age (years)*	64.7 (12.9)	67.4 (10.9)	65.7 (13.0)	58.6 (11.2)	64.9 (12.4)
Follow-up period	40.2 (35.6)	26.1 (23.5)	17.8 (14.9)	8.1 (7.9)	21.4 (17.7)
(months) *					
Survival					
Yes	38	18	22	4	82
No	7	27	32	20	86

* Age and follow-up period are mean (S.D.).

### Nuclear SOX4 Expression was Upregulated and Associated with Clinicopathologic Parameters in GC

**Figure 1:**
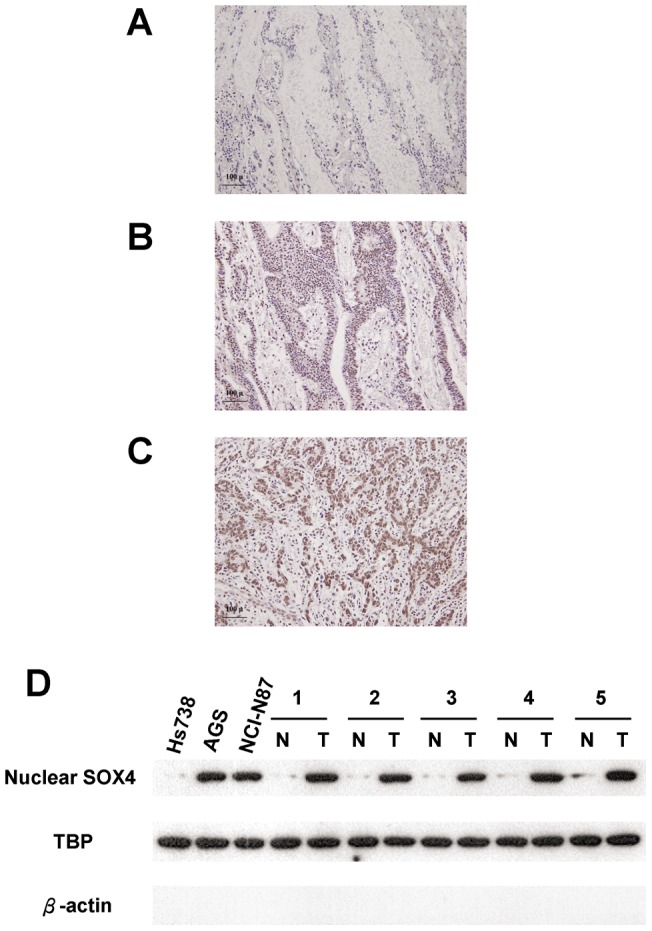
Expression of SOX4 in gastric tissues and cell lines. Panels A to C. GC specimens analyzed by immunohistochemistry with an antibody against SOX4. The staining of SOX4 is observed in the nucleus and is very weak in the cytoplasm. **Panel A** shows a non-tumor sample without nuclear SOX4 expression; **Panel B** shows a tumor sample with low expression level of nuclear SOX4; **Panel C** shows a tumor sample with high expression level of nuclear SOX4. **Panel D:** Nuclear SOX4 protein expression was examined in 3 gastric cells and 5 non-tumor/tumor pairs of gastric tissues. Magnification: 200×.

We used immunohistochemical analysis to investigate the expression of nuclear SOX4 in tissues obtained from our study patients (Figures 1A to C). Nuclear SOX4 expression was significantly higher in tumor tissues than in non-tumor tissues (*P*<0.001). Overexpression of nuclear SOX4 (scores of 2 or 4) was observed in 90 of the 168 patients (53.5%). Western blot analysis also demonstrated that the expression of SOX4 was substantially increased in gastric cancer cells and tissues when compared with normal cells and tissues (Figure 1D). Additionally, quantitative real-time PCR analysis demonstrated that the expression of SOX4 mRNA was substantially increased in tumor tissues when compared with non-tumor tissues (Table 2). As shown in Table 3, overexpression of nuclear SOX4 correlated significantly with the following parameters: depth of invasion (*P*<0.0001), nodal status (*P* = 0.0055), distant metastasis (*P* = 0.0195), stage (*P* = 0.0003), and vascular invasion (*P* = 0.0383). No significant association emerged between overexpression of nuclear SOX4 and age or gender.

**Table 2:** Quantification of SOX4 mRNA expression by quantitative real-time PCR in 10 tumor and non-tumor pairs of gastric tissues.

	Non-tumor			Tumor		
No.	SOX4	β-actin	Δ*C_non-tumor_*	SOX4	β-actin	Δ*C_tumor_*
S0059	29.87	19.95	9.92	30.19	24.57	5.62
S0225	33.26	24.28	8.98	31.46	26.37	5.09
S0428	30.14	19.91	10.23	30.84	23.99	6.85
S0438	27.51	19.16	8.35	28.67	23.46	5.21
S0706	31.29	22.88	8.41	31.86	25.58	6.28
S0735	29.76	21.17	8.59	30.84	24.51	6.33
S0891	33.3	23.45	9.85	32.16	26.55	5.61
S1357	30.52	20.77	9.75	31.07	25.13	5.94
S1944	34.96	24.49	10.47	32.88	26.04	6.84
S2089	30.82	22.18	8.64	31.15	25.74	5.41

**Table 3:** Nuclear SOX4 expression in GC and its correlation with clinicopathologic parameters.

		Nuclear SOX4 expression		
		Score = 0 or 1	Score = 2 or 4	
Variable	n	(n = 78)	(n = 90)	*P* *
Age (yr)				0.1403
≥66	77	31	46	
<66	91	47	44	
Gender				0.3872
Male	104	51	53	
Female	64	27	37	
Depth of invasion				<0.0001
T1	26	23	3	
T2	55	16	39	
T3	74	34	40	
T4	13	5	8	
Nodal status				0.0055
N0	73	44	29	
N1	29	14	15	
N2	28	8	20	
N3	38	12	26	
Distant metastasis				0.0195
Absent	155	76	79	
Present	13	2	11	
Stage				0.0003
I	45	28	17	
II	45	26	19	
III	54	21	33	
IV	24	3	21	
Vascular invasion				0.0383
Absent	100	53	47	
Present	68	25	43	

* All statistical tests were two-tailed and the significance level was *P*<0.05.

### Overexpression of Nuclear SOX4 As a Prognostic Marker for GC

**Figure 2:**
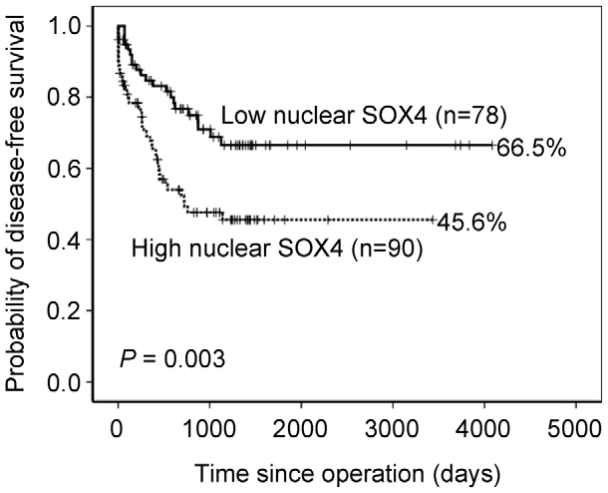
Disease-free survival analysis for 168 patients, stratified by nuclear SOX4 immunoreactivity (low nuclear SOX4: score = 0 or 1; high nuclear SOX4: score  = 2 or 4). All statistical tests were two-tailed and the significance level was *P*<0.05.

**Figure 3:**
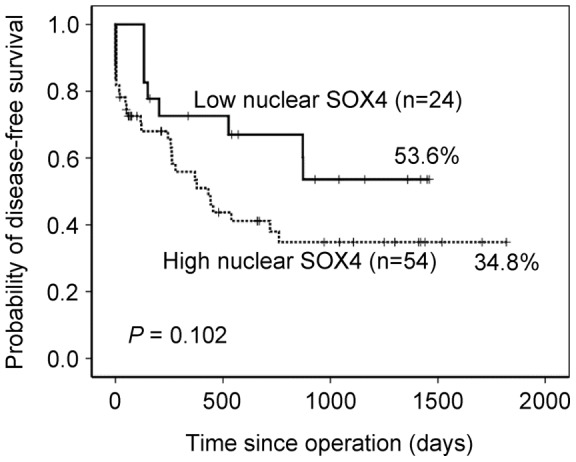
Disease-free survival analysis for 79 high-stage GC patients, stratified by nuclear SOX4 immunoreactivity (low nuclear SOX4: score  = 0 or 1; high nuclear SOX4: score  = 2 or 4). All statistical tests were two-tailed and the significance level was *P*<0.05.

Correlations of clinical outcomes with nuclear SOX4 expression are shown in Figure 2. Overexpression of nuclear SOX4 was significantly associated with shorter disease-free survival (*P* = 0.003). Patients with high expression levels of nuclear SOX4 achieved a 5-year disease-free survival rate of 45.6% compared with 66.5% for patients with low expression levels. Furthermore, high-stage GC (stage III and IV) was used to find out the effect of nuclear SOX4 overexpression on the prognosis. However, the association between overexpression of nuclear SOX4 and disease-free survival was only borderline significant (*P* = 0.102, Figure 3).

The results of univariate analysis of the prognostic markers of GC are shown in Table 4. Disease-free survival was significantly correlated with each of the following: nodal status (*P*<0.001), distant metastasis (*P*<0.001), stage (*P*<0.001), vascular invasion (*P*<0.001), and overexpression of nuclear SOX4 (*P* = 0.004). However, the association between overexpression of nuclear SOX4 and survival was not significant after controlling for other well-known prognostic markers in multivariate analysis (*P* = 0.186, Table 5). In multivariate analysis, depth of invasion (Hazard Ratio (HR)  = 2.091, 95% Confidence Interval (CI)  = 1.073 to 4.077, *P* = 0.030), nodal status (HR = 3.901, 95% CI = 1.589 to 9.580, *P* = 0.003), distant metastasis (HR = 15.453, 95% CI = 6.419 to 37.114, *P*<0.001) and vascular invasion (HR = 1.849, 95% CI = 1.058 to 3.229, *P* = 0.031) were prognostically independent.

**Table 4:** Univariate analysis of prognostic markers in 168 patients with GC.

Variable	HR (95% CI)*	*P* *
Depth of invasion	1.554 (0.942–2.562)	0.084
T1 + T2		
T3 + T4		
Nodal status	3.773 (2.077–6.853)	<0.001
N0		
N1 + N2 + N3		
Distant metastasis	15.591 (7.603–31.971)	<0.001
Absent		
Present		
Stage	2.837 (1.677–4.736)	<0.001
I + II		
III + IV		
Vascular invasion	3.305 (1.986–5.499)	<0.001
Absent		
Present		
Nuclear SOX4	2.158 (1.276–3.649)	0.004
Low expression		
High expression		

* All statistical tests were two-tailed and the significance level was *P*<0.05. HR  =  hazard ratio; CI =  confidence interval.

**Table 5:** Multivariate analysis of prognostic markers in 168 patients with GC.

Variable	HR (95% CI)*	*P* *
Depth of invasion	2.091 (1.073–4.077)	0.030
T1 + T2		
T3 + T4		
Nodal status	3.901 (1.589–9.580)	0.003
N0		
N1 + N2 + N3		
Distant metastasis	15.453 (6.419–37.114)	<0.001
Absent		
Present		
Stage	0.493 (0.200–1.213)	0.124
I + II		
III + IV		
Vascular invasion	1.849 (1.058–3.229)	0.031
Absent		
Present		
Nuclear SOX4	1.451 (0.835–2.521)	0.186
Low expression		
High expression		

* All statistical tests were two-tailed and the significance level was *P*<0.05.

## Discussion

Gastric cancer remains a major public health problem worldwide [1]. Surgical resection is generally considered the best treatment to improve the prognosis when early diagnosis of GC is successful [29]. Unfortunately, most cases of GC are diagnosed late, at a locally advanced stage. Patients with advanced tumors often undergo radical gastrectomy, which leads to a high level of morbidity and does not lessen the risk of recurrence [30]. Greater knowledge of the molecular mechanisms underlying the development of this deadly neoplasm is required if novel strategies to prevent and treat GC are to be developed. In particular, identification of molecules that are altered during cancer initiation and progression can provide valuable tools as prognostic markers or therapeutic targets.

The expression of SOX4 in human cancers varies according to cancer type. The SOX4 level is elevated in numerous human cancers, including of the bladder, prostate, endometrium, and liver, whereas it is decreased in melanoma and gallbladder cancer [20], [21], [31]–[34]. Our literature review identified only one study that had investigated the expression of SOX4 in human GC. This study, by Shen et al., showed that SOX4 was overexpressed in GC patients [35]. In the present study, we assessed the expression levels of nuclear SOX4 in gastric tissues obtained from 168 patients with GC. Our results were consistent with those of Shen et al. and showed that nuclear SOX4 expression was elevated in gastric tumor tissues relative to non-tumor gastric tissues. The immunoblotting results confirmed that nuclear SOX4 expression was higher in GC cells than in normal gastric cells.

Our findings also showed that overexpression of nuclear SOX4 in GC tissues was closely correlated with tumor invasion and metastasis. The mechanism by which SOX4 exerts its invasive and metastatic activity remains unclear. In the first line of evidence, miRNAs (small noncoding RNAs with regulatory functions) were shown to be associated with tumor invasion and metastasis [36]–[38]. Previous work by Tavazoie et al. showed that miR-335 suppresses metastasis through down-regulation of SOX4 [26]. This result suggested that SOX4 is linked to tumor aggressiveness.

The second line of evidence has been provided by studies on epithelial-mesenchymal transition (EMT), which is a key step during embryogenesis [39]. Accumulating evidence suggests that inappropriate utilization of EMT might be a component of the invasion of many tumors of epithelial tissues. Cell characteristics are strongly affected during EMT, resulting in alterations to cell-cell and cell-matrix interactions, cell motility, and invasiveness [40], [41]. Recent study of Zhang et al. showed that overexpression of SOX4 in human mammary epithelial cells led to the acquisition of mesenchymal traits, and enhanced cell migration and invasion. Furthermore, SOX4 positively regulated the expression of known EMT inducers and activated the TGF-β pathway to contribute to EMT. The expression of SOX4 was induced by TGF-β and was necessary for TGF-β-induced EMT. These findings show that SOX4 plays an important role in the progression of breast cancer, by orchestrating EMT [42]. These studies may account in part for the association of overexpression of nuclear SOX4 with tumor invasion and metastasis.

Precise prediction of the risk of recurrence would assist in minimizing the adverse effects of GC and maximizing the therapeutic effect of treatment. Of the available prognostic markers for GC, the AJCC stage is most important. However, the prognosis varies even among patients at the same disease stage; hence, alternative prognostic markers are sought. Few studies have investigated the prognostic value of SOX4 proteins. Jafarnejad et al. showed that in melanoma patients, a strong association existed between reduced SOX4 expression and poor patient survival [33]. Similarly, Kim et al. showed that overexpression of SOX4 protein in patients with hepatocellular carcinoma was associated with improved patient outcomes [32]. In addition, Aaboe et al. showed that a strong association existed between increased SOX4 expression and increased patient survival in cases of bladder cancer [20]. We found no published reports discussing the prognostic significance of SOX4 in human GC. The results of this study showed that nuclear SOX4 overexpression was inversely correlated with patient survival; this finding contradicted the previously reported positive correlations. Our study was the first to show that overexpression of nuclear SOX4 can predict poorer outcomes for patients with GC. Overexpression of nuclear SOX4 appears to be a useful marker to predict outcomes in patients with GC who have received surgical resection of the tumor. Thus, patients with GC who display overexpression of nuclear SOX4 should be followed up carefully. Because our patient group was small, future studies should include a larger GC patient group to elucidate the prognostic significance of nuclear SOX4 in this disease.

In summary, this study provided evidence for the clinical significance of overexpressed SOX4 in patients with GC. Our findings indicate that targeting SOX4 might provide a new therapeutic modality for the treatment of GC.
